# Regulation of the microprocessor by post-translational modifications

**DOI:** 10.3389/fcell.2025.1721087

**Published:** 2025-12-01

**Authors:** Ka Weng Leong, Mark M. W. Chong

**Affiliations:** 1 RNA and T cell Biology, St Vincent’s Institute of Medical Research, Fitzroy, VIC, Australia; 2 Department of Medicine, University of Melbourne, Parkville, VIC, Australia

**Keywords:** microprocessor, DROSHA, DGCR8, microRNA biogenesis, phosphorylation, acetylation, ubiquitination, SUMOylation

## Abstract

The Microprocessor is an essential protein complex that is responsible for the first processing step in the biogenesis of canonical microRNAs. The core of this complex is composed of two proteins, the ribonuclease III enzyme DROSHA and its double-stranded RNA-binding cofactor DGCR8. Dysregulation of the expression of the Microprocessor contributes to many disorders, including pluripotency defects, immune dysfunction, cancers, and neurological diseases. Multiple post-translational modifications (PTMs) have been reported for DROSHA and DGCR8, and these are thought to play roles in regulating Microprocessor levels and its functions; however, most of these PTMs remain functionally uncharacterized. In this review, we discuss these PTMs of the Microprocessor, focusing on phosphorylation, acetylation, ubiquitination, and SUMOylation, and how these modifications are thought to regulate protein stability, microRNA production, and other non-canonical Microprocessor activities.

## 1 Introduction

MicroRNAs (miRNAs) are a class of small non-coding RNAs that play a critical role in gene regulation. They form complementary base-pairs with protein-coding messenger RNA (mRNA) targets, which lead to the degradation and translation repression of mRNAs (Bartel, 2018; Kim et al., 2025). Each miRNA can target multiple mRNAs (Lim et al., 2005), and collectively, miRNAs are predicted to regulate more than half of the protein-coding genes in the genome (Friedman et al., 2009). Tight regulation of miRNA biogenesis is crucial for maintaining the normal physiological state of cells, and dysregulation of miRNAs is associated with many diseases, such as developmental defects, autoimmune disorders, and cancer (Bartel, 2018; Chen et al., 2016; Garzon et al., 2006; Jeker et al., 2013; Volinia et al., 2006).

**FIGURE 1:**
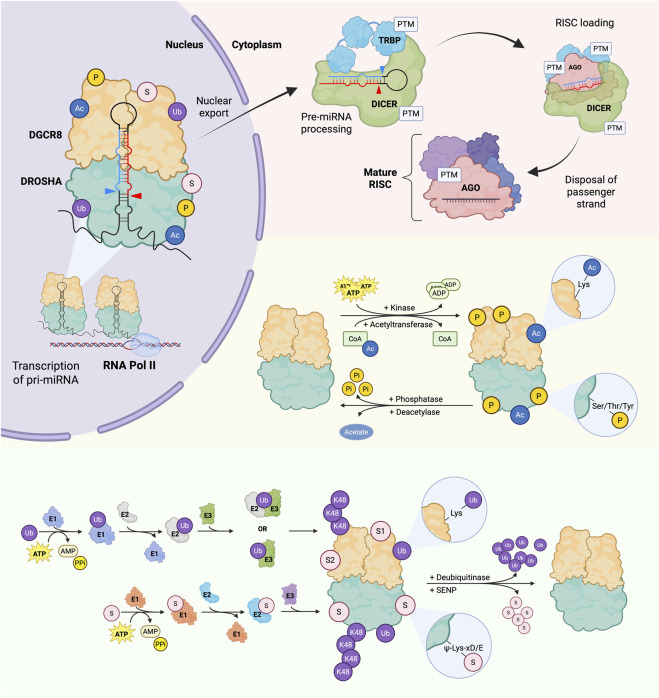
Post-translational modifications regulate the miRNA biogenesis machinery. Pri-miRNAs are transcribed by RNA polymerase II (Pol II) and co-transcriptionally processed by the Microprocessor complex, composed of DROSHA and DGCR8. The complex recognises and releases the stem-loop pre-miRNA intermediate, which is then exported to the cytoplasm. There, another RNase III protein, DICER, together with TRBP, removes the apical loops and loads one strand (the mature miRNA) into an AGO-containing RNA-induced silencing complex (RISC) to mediate mRNA degradation or translational repression. Components of the miRNA machinery, including DROSHA, DGCR8, TRBP, DICER, and AGO, are all regulated by post-translational modifications (PTMs). Summary of the enzymatic reactions that add/remove the four PTMs found on Microprocessor components. Kinases transfer a phosphate group (P) from ATP to serine, threonine, or tyrosine residues of target proteins, whereas phosphatases reverse the modification. Acetyltransferases transfer an acetyl group (Ac) from coenzyme A (CoA) to lysine residues of target proteins, while deacetylases reverse the modification. Ubiquitin-activating enzymes (E1), ubiquitin-conjugating enzymes (E2), and ubiquitin ligases (E3) sequentially facilitate the transfer of ubiquitin (Ub) to lysine residues of target proteins. Ubiquitin itself can be ubiquitinated, forming differently linked ubiquitin chains, such as K48-linked chains. Ubiquitination is reversed by deubiquitinases. SUMO-activating enzymes (E1), SUMO-conjugating enzymes (E2), and SUMO ligases (E3) successively catalyse the transfer of small ubiquitin-like modifier (SUMO) to lysine residues within the SUMOylation consensus motif ψKxD/E, where ψ is a large hydrophobic amino acid, x is any amino acid, D and E are acidic amino acids (aspartic acid and glutamic acid, respectively). Only SUMO1 (S1) and SUMO2 (S2) are shown in the illustration, while multiple SUMO isoforms exist. SUMO-specific proteases (SENPs) remove SUMO from SUMO-modified proteins. Created in BioRender (https://BioRender.com/98kt2ep).

In canonical miRNA biogenesis, the primary (pri)-miRNAs are transcribed and co-transcriptionally processed by the Microprocessor complex (Morlando et al., 2008) (Figure 1A). It is minimally composed of the ribonuclease III (RNase III) protein DROSHA, and its double-stranded (ds)RNA-binding partner, DGCR8 (Nguyen et al., 2015). The complex recognizes and cleaves miRNA-containing stem-loop structures embedded within the long pri-miRNA transcripts (Morlando et al., 2008; Yin et al., 2015). These stem-loops, or pre-miRNAs, are then exported to the cytoplasm and further processed by another RNase III protein, DICER (Lee et al., 2002). The resulting miRNA duplex is loaded onto an argonaute (AGO) protein, the core component of the RNA-induced silencing complex (RISC), where one strand is retained as the mature miRNA (Kawamata and Tomari, 2010).

In addition to microRNA biogenesis, the Microprocessor has also been reported to exert other molecular functions. These include recognizing stem-loop-containing viral RNAs (Aguado et al., 2017), binding or cleaving stem-loop-embedded protein-coding mRNAs (Cirera-Salinas et al., 2017; Han et al., 2009; Havens et al., 2014; Johanson et al., 2015; Knuckles et al., 2012; Lee et al., 2017; Marinaro et al., 2017; Rolando et al., 2016), and interacting with DNA repair proteins (Cabrini et al., 2021; Calses et al., 2017; Hang et al., 2021; Lu et al., 2018). Through these activities, the Microprocessor contributes to a range of other biological processes, including anti-viral defense (Aguado et al., 2017), autoregulation (Han et al., 2009; Lee et al., 2017), cell fate determination (Johanson et al., 2015; Knuckles et al., 2012; Marinaro et al., 2017; Rolando et al., 2016), alternative splicing (Cirera-Salinas et al., 2017; Havens et al., 2014; Lee et al., 2017), and DNA damage repair (Cabrini et al., 2021; Calses et al., 2017; Hang et al., 2021; Lu et al., 2018).

PTMs are the covalent addition or removal of modifying groups on the side chain of amino acid residues (Ramazi and Zahiri, 2021), and they can alter the chemical and physical properties of proteins, thereby regulating their subcellular localization, structures, stability, and functions (Ryšlavá et al., 2013). A growing number of PTMs have been reported for the miRNA machinery and their associated proteins, suggesting that PTMs are an important mechanism of regulating the miRNA pathway (Ha and Kim, 2014) (Figure 1A). This includes ERK phosphorylation and stabilization of TRBP, the cofactor of DICER, which promotes mature miRNA production (Paroo et al., 2009). Another is the SUMO2/3 modification of DICER, which downregulates protein levels and its processing of pre-miRNAs (Gross et al., 2014). Both Microprocessor proteins, DROSHA and DGCR8, also appear to be heavily modified by phosphorylation, acetylation, ubiquitination, and SUMOylation. DROSHA is also reportedly methylated at a single residue, R271, which was identified by high-throughput discovery mass spectrometry, but its functional relevance remains unknown (Larsen et al., 2016). In this review, we will summarize the reported phosphorylation, acetylation, ubiquitination, and SUMOylation of DROSHA and DGCR8 and discuss how these PTMs regulate the diverse functions of the Microprocessor (Table 1).

**TABLE 1:** PTMs identified on human DROSHA and DGCR8.

Modification	Position	Enzyme	Molecular effect	References
DROSHA: PTMs detected only via high-throughput proteomic discovery mass spectrometry				
Methylation (mono)	R271	Unknown	Unknown	Larsen et al. (2016)
Phosphorylation	Y228, S237, S267, Y269, S276, S317, S357, S373, S491, S698, Y705, T747, S796, S807, S1293	Unknown	Unknown	Beli et al. (2012), Christensen et al. (2010), Franz-Wachtel et al. (2012), Hornbeck et al. (2015), Grosstessner-Hain et al. (2011), Kettenbach et al. (2011), Klammer et al. (2012), Mertins et al. (2013), Mertins et al. (2014), Mertins et al. (2016), Olsen et al. (2010), Pan et al. (2009), Rigbolt et al. (2011), Santamaria et al. (2011), Schweppe et al. (2013), Sharma et al. (2014), Shiromizu et al. (2013), Sui et al. (2008), Weber et al. (2012), Zhou et al. (2013)
SUMOylation	K388, K409	Unknown	Unknown	Hendriks et al. (2014)
Ubiquitination	K382, K409, K447, K738, K748, K792, K795, K809, K827, K831, K1207, K1250, K1262, K1277, K1285, K1313, K1341, K1353, K1373	Unknown	Unknown	Akimov et al. (2018), Boeing et al. (2016), Udeshi et al. (2013), Wagner et al. (2011)
DROSHA: Experiment-validated PTMs				
Acetylation	Unknown	p300, HDAC1*	Unknown	Wada et al. (2012)
	K382	p300, CBP, GCN5	Protein stabilization	Tang et al. (2013)
	N-terminus			
Phosphorylation	S221, S255, T274, S300, S355	CDK5, P38A	Molecular association, cytoplasmic localization, protein degradation	Huang et al. (2024), Yang et al. (2015)
	S300, S302	GSK3β	Nuclear localization	Tang et al. (2011), Tang et al. (2010)
K48-linked Ubiquitination	N-terminus	Unknown	Protein degradation	Tang et al. (2013)
Ubiquitination	Unknown	MDM2	Protein degradation, miRNA processing	Ye et al. (2015)
DGCR8: PTMs detected only via high-throughput proteomic discovery mass spectrometry				
Phosphorylation	S6, S8, S339, S367, S368, S385, S545, T547, S711, S714	Unknown	Unknown	Hornbeck et al. (2015), Kettenbach et al. (2011), Mertins et al. (2013), Mertins et al. (2014), Mertins et al. (2016), Schreiber et al. (2010), Sharma et al. (2014), Stuart et al. (2015), Van Hoof et al. (2009), Zhou et al. (2013)
Ubiquitination	K113, K134, K380, K424, K431, K474, K488, K582, K640, K647, K659, K713, K726	Unknown	Unknown	Akimov et al. (2018)
DGCR8: Experiment-validated PTMs				
Acetylation	K561, K562, K565	p300, HDAC1*	miRNA processing	Wada et al. (2012)
Phosphorylation	S35, T42, S59, S92, S95, S109, S123, T125, S153, S156, Y267, S271, S275, T279, S280, T371, S373, S377, S383, S385, S397, S434, S493	ERK	Protein stabilization, miRNA processing, mRNA alternative splicing	Cirera-Salinas et al. (2017), Herbert et al. (2013)
	S95, S271, S275, T371, S373, S377, S434, S619	Unknown	Unknown	Calses et al. (2017)
	S153	JNK1	Unknown	Calses et al. (2017)
	Y267	ABL	miRNA processing	Tu et al. (2015)
	S677	ATM	Molecular association, protein stabilization	Hang et al. (2021)
SUMOylation	K259	SUMO1	Nuclear retention	Zhu et al. (2016)
	K707	SUMO1	Protein stabilization, miRNA-binding affinity	Zhu et al. (2015)
	K259, K426, K707	SUMO2	miRNA processing, proliferation	Li et al. (2023)
K48-linked ubiquitination	Unknown	USP51^	Protein degradation	Hang et al. (2021)

*deacetylase and ^deubiquitinase.

## 2 Implications of microprocessor dysregulation

DROSHA is a nuclear RNase III that contains two tandem RNase III domains (RIIIDs), RIIIDa and RIIIDb, followed by a dsRNA binding domain (dsRBD) at the C-terminus (Kwon et al., 2016). The N-terminal extension comprises a proline-rich (P-rich) domain and an arginine/serine-rich (R/S-rich) domain, followed by a central domain (CED) (Kwon et al., 2016). DGCR8 is a nuclear dsRNA-binding protein composed of an N-terminal tail, an RNA-binding heme domain (Rhed), followed by two dsRBDs and the C-terminal tail (CTT) (Senturia et al., 2010). The DROSHA dsRBDs alone have weak affinity for dsRNAs and require a dimer of DGCR8 for RNA recruitment and substrate recognition (Kranick et al., 2017; Roth et al., 2013; Wostenberg et al., 2010). DGCR8 stabilizes DROSHA by binding DROSHA RIIIDs with its CTT, while DROSHA controls DGCR8 expression by cleaving DGCR8 mRNA (Han et al., 2009; Nguyen et al., 2015).

Given the molecular functions of the Microprocessor, unsurprisingly, dysregulation of either DROSHA or DGCR8 can result in adverse consequences. Both proteins are critical for early embryogenesis. DROSHA or DGCR8 deficiency in mice results in impaired embryonic stem cell differentiation and lethality soon after implantation (Deng et al., 2019; Lyu et al., 2024; Wang et al., 2007). Loss of DROSHA (Chong et al., 2008) or DGCR8 (Jeker et al., 2013) disrupts miRNA production and impairs regulatory T cells homeostasis and thus is suggested to drive immune disorders. In humans, the loss of one functional allele of *DGCR8* is sufficient to impair miRNA production, suggesting to be the cause of the physiological defects seen in 22q11.2 deletion syndrome, including pluripotency defects, immunodeficiency due to an underdeveloped thymus, and psychiatric illnesses (Colomer-Boronat et al., 2025; McDonald-McGinn et al., 2015).

Mutations in *DGCR8* are observed in thyroid cancer (Paulsson et al., 2021; Rodrigues et al., 2024; Rodrigues et al., 2022), and mutations in both *DROSHA* and *DGCR8* occur in Wilms’ tumor (Rakheja et al., 2014; Walz et al., 2015), highlighting the importance of maintaining Microprocessor integrity. While dysregulated expression of these Microprocessor components or the mislocalization of DROSHA have been reported in a range of other cancers (Huang et al., 2014; Lin and Gregory, 2015; Theotoki et al., 2025).

Neurological disorders can arise from abnormal intracellular localization or downregulation of Microprocessor components. In fragile X-associated tremor/ataxia syndrome, DROSHA and DGCR8 are sequestered by nuclear RNA aggregates (Sellier et al., 2013). While in amyotrophic lateral sclerosis, DROSHA is mislocalized into the cytoplasm and forms aggregates with the FUS mutant (Kour et al., 2023). In traumatic brain injury, phosphorylation of DROSHA is associated with downregulated expression, which drives neuronal apoptosis and further exacerbates the condition (Huang et al., 2024). DGCR8 deficiency is implicated in the impaired cognition and schizophrenia observed in 22q11.2 deletion syndrome (Fénelon et al., 2011; Ouchi et al., 2013; Rao et al., 2025).

These are examples of the consequences of the most severe lesions of DROSHA and DGCR8 deficiency. However, more nuanced phenotypes are likely to occur due to defective PTMs of these proteins.

## 3 Post-translational modifications on the microprocessor

### 3.1 Phosphorylation

Phosphorylation of a protein is the reversible transfer of a phosphoryl group (PO_3_^2-^) from an adenosine triphosphate (ATP) to a specific residue by kinases, most commonly to the hydroxyl group-containing serine, threonine, and tyrosine (Ardito et al., 2017) (Figure 1B). This modification is reversed by phosphatases (Ardito et al., 2017). The addition of a phosphoryl group increases the polarity and hydrophilicity of a protein, affecting conformation and substrate affinity, and thus the functions of the protein (Ardito et al., 2017).

The R/S-rich domain in the N-terminal of DROSHA provides an extensive platform for phosphorylation events, with 14 out of 21 phosphosites documented in PhosphoSitePlus v6.8.1 located in this domain (Hornbeck et al., 2015). Glycogen synthase kinase 3 beta (GSK3β) and Polo-Like Kinase 1 (PLK1) have been reported to phosphorylate DROSHA at S300 and S302, which promotes its nuclear translocation, its association with DGCR8, and the downstream miRNA production (Fletcher et al., 2023; Tang et al., 2011; Tang et al., 2010). p38 mitogen-activated protein kinase (MAPK) reportedly phosphorylates another group of residues, S221, S255, T274, S300, and S355 in DROSHA, upon cellular stress (Yang et al., 2015). This drives its dissociation from DGCR8, translocation to the cytoplasm, and ultimately degradation by calpain (Yang et al., 2015). The consequence is a reduction in the miRNA biogenesis, leading to apoptosis (Yang et al., 2015). Interestingly, these examples demonstrate that the phosphorylation of S300 can facilitate counterposed translocations, though the nuclear export of DROSHA additionally requires the phosphorylation of several other residues in the domain. Besides p38 MAPK, CDK5 has also been reported to phosphorylate S221, S255, T274, S300, and S355 in DROSHA after traumatic brain injury, leading to its degradation by calpain and cell apoptosis (Huang et al., 2024).

A phosphoproteomic analysis of overexpressed human DGCR8 in insect or human cell lines identified 23 phosphorylated sites, with 5 of these sites phosphorylated only in the insect cells (Herbert et al., 2013). Mutating all 23 sites at once revealed that DGCR8 can be phosphorylated by extracellular signal-regulated kinase (ERK), which contributes to the stabilization of the protein, pro-growth miRNA production, and enhanced cell proliferation (Zhu et al., 2015). The phosphorylation of the 23 sites was later also reported to be important for controlling the alternative splicing of *Tcf7l1* mRNA, and this serves as a checkpoint for stem cell differentiation (Cirera-Salinas et al., 2017). However, which specific phosphosites are important remained ambiguous. A separate study showed that epidermal growth factor-induced ERK activation also results in DGCR8 phosphorylation (Zhu et al., 2015). This phosphorylates S109, S153, T371, and S377, and was predicted to be responsible for promoting DGCR8 SUMOylation (Zhu et al., 2015). Concordantly, coilin, a nuclear Cajal bodies structural protein, was suggested to enhance S377 phosphorylation, which in turn promotes DGCR8 stability and the downstream miRNA biogenesis (Lett et al., 2021).

The phosphorylation of specific sites in DGCR8 were also reported to regulate DNA damage repair pathways in either a miRNA-dependent or a miRNA-independent manner. DNA repair mechanisms are crucial for preventing the accumulation of mutations and cellular transformation. The phosphorylation at S153 by Jun N-terminal kinase (JNK) confers resistance to mammalian cells against ultraviolet radiation through the transcription-coupled nucleotide excision repair (TC-NER) pathway (Calses et al., 2017). Upon ionizing radiation, DGCR8 S677 is phosphorylated by the kinase ATM, allowing it to be recognized by USP51, which enhances its protein stabilization (Hang et al., 2021). This phosphorylated DGCR8 can then contribute to double-stranded break repair by recruiting RNF168 to RNF8 and MDC1 for histone ubiquitination (Hang et al., 2021). The function of the phosphorylated DGCR8 in DNA repair after ultraviolet or ionizing radiation is independent of both DROSHA and miRNA (Calses et al., 2017; Hang et al., 2021). The tyrosine kinase ABL was also reported to phosphorylate DGCR8 at Y267, potentially in a tissue-specific manner, to reinforce the interaction between DGCR8 and its RNA substrate for the production of DROSHA-dependent miR-34c, which is involved in DNA repair after DNA damage induced by cisplatin treatment (Tu et al., 2015). These together suggest that proper phosphorylation of DGCR8 prevents mutation accumulation and thus suppresses cancer development.

### 3.2 Acetylation

Acetylation, the addition of an acetyl group (CH_3_CO^−^) to a residue, is mediated by acetyltransferases and removed by deacetylases (Narita et al., 2019) (Figure 1C). Although acetylation occurs more frequently on lysine residues, it can also occur on the N-terminal amino acid and on serine, threonine, and tyrosine residues (Xia et al., 2020). The addition of an acetyl group to lysine neutralizes the positive charge and thus can alter the structure, stability, and function of a protein (Narita et al., 2019). Moreover, acetylation often competes with ubiquitination for the same lysine residues (Narita et al., 2019).

DROSHA contains 13 lysine residues at the N-terminus that can undergo acetylation or ubiquitination to regulate protein stability (Tang et al., 2013). DROSHA was reported to be weakly acetylated by p300 and this can be completely removed by HDAC1, a histone deacetylase that co-immunoprecipitates with the Microprocessor in HEK293 cells and K562 cells (Wada et al., 2012). More specifically, DROSHA was reported to be acetylated at K382 and other lysine residues by several histone acetyltransferases, including p300, CBP, and GCN5, and this acetylation is enhanced upon inhibition of HDAC-mediated deacetylation, resulting in DROSHA protein stabilization (Tang et al., 2013).

DGCR8 is acetylated in its native form (Wada et al., 2012). It can be further acetylated by p300 and can be almost fully deacetylated by HDAC1 (Wada et al., 2012). All three conserved and indispensable lysine residues K561, K562, and K565 in the dsRBD of DGCR8 can be deacetylated by HDAC1, which enhances the affinity of DGCR8 for primary miRNA substrates and thus regulates the production of miRNAs (Acevedo et al., 2015; Kranick et al., 2017; Wada et al., 2012; Wostenberg et al., 2010). This suggests HDAC1 exerts dual effects on gene silencing, through conventional histone deacetylation and chromatin condensation to suppress transcription, as well as enhanced miRNA production via the non-canonical DGCR8 deacetylation for post-transcriptional repression (Dunaway and Pollock, 2022; Wada et al., 2012).

### 3.3 Ubiquitination

Ubiquitination is the reversible process of the addition of ubiquitin molecules to the side chain of an amino acid residue by a protein complex containing ubiquitin-activating enzyme (E1), ubiquitin-conjugating enzyme (E2), and ubiquitin ligase (E3) (Damgaard, 2021) (Figure 1D). Ubiquitin is a small peptide of 76 amino acids that acts as a tag on proteins for the regulation of several cellular processes, including cell signaling, protein trafficking, and DNA repair, either via proteolytic or non-proteolytic mechanisms (Damgaard, 2021). Like acetylation, ubiquitination can occur on multiple amino acids but more frequently occurs at lysine, which, as mentioned above, can compete with acetylation (Kelsall, 2022). Apart from the targeted proteins, ubiquitin itself can also be ubiquitinated on any of the seven internal lysine residues, K6, K11, K27, K29, K33, K48, and K63, forming different lysine-linked chains of ubiquitin (Damgaard, 2021). The linkage affects the conformation of the poly-ubiquitin chains and thus signals different downstream pathways (Damgaard, 2021). For example, K48-linked polyubiquitination canonically functions as a tag for protein degradation by the 26S proteasome, while K63-linked polyubiquitination typically contributes to endocytosis and cellular signaling (Sun and Zhang, 2022; Wang et al., 2012).

Human DROSHA contains 70 lysine residues, of which ubiquitination was detected at 21 by high-throughput proteomic discovery mass spectrometry (Hornbeck et al., 2015). DROSHA protein stability is regulated via the K48-linked ubiquitin-proteasome pathway, with one or more of the 13 lysine residues in the DROSHA N-terminus being the potential ubiquitination target (Tang et al., 2013). mTOR activation indirectly reduces DROSHA protein level through ubiquitin-mediated degradation and drives global miRNA reduction (Ye et al., 2015). The activation upregulates the E3 ubiquitin ligase MDM2 in p53-dependent and -independent manners, and MDM2 then ubiquitinates DROSHA for proteasome-mediated degradation (Ye et al., 2015). This results in the reduction of specific miRNAs, including miR-297 and miR-567, to drive apoptosis (Ye et al., 2015). Whereas the deprivation of either amino acids or glucose reduces mTOR activation, resulting in reduced MDM2 level, stabilization of DROSHA, and thus improved survival (Ye et al., 2015). These suggest DROSHA plays a protective role under nutrient- and energy-restricted conditions and restricts cell overgrowth when there is sufficient resource.

Similarly, DGCR8 protein stability is regulated by the K48-linked ubiquitin-proteasomal pathway. DGCR8 contains 61 lysine residues. Although the basal level of DGCR8 ubiquitination was barely detectable (Li et al., 2023; Zhu et al., 2015), 13 of the lysine residues were reported to be ubiquitinated (Hornbeck et al., 2015). The proteasome inhibitor MG132, but not the lysosome inhibitor chloroquine, could block the degradation of DGCR8 (Zhu et al., 2015). Concordantly, as previously mentioned, DGCR8 is phosphorylated at S677 upon ionizing radiation for its recognition by USP51, a deubiquitinase, to remove the K48-linked ubiquitin for its stabilization (Hang et al., 2021).

### 3.4 SUMOylation

SUMOylation is another reversible process in which a small ubiquitin-like modifier (SUMO) molecule is covalently added to proteins, usually at a lysine residue embedded within a consensus motif (Celen and Sahin, 2020) (Figure 1E). The conjugatable SUMO family in mammals includes SUMO1, SUMO2, and SUMO3, which are around 100 amino acids and are structurally similar to ubiquitin (Han et al., 2018; Seeler and Dejean, 2003; Wang and Matunis, 2023). SUMO1 shares approximately 50% sequence identity with SUMO2 and SUMO3, while SUMO2 and SUMO3 are around 97% identical, making them difficult to discriminate from each other via antibody detection and thus are often referred to as SUMO2/3 (Pichler et al., 2017). SUMO proteins serve as regulatory tags that modulate target proteins for protein localization, stability, and molecular interactions, with SUMO1 primarily functioning under normal physiological conditions and SUMO2/3 under stressed conditions (Han et al., 2018; Seeler and Dejean, 2003). Like ubiquitination, SUMOylation is catalyzed by a cascade of enzymes, including a SUMO-activating enzyme (E1), a SUMO-conjugating enzyme (E2), and a SUMO ligase (E3), with the removal catalyzed by SUMO-specific proteases (SENPs) (Han et al., 2018). SUMOylation can be induced by phosphorylation and in turn can also affect other subsequent PTMs, including acetylation and ubiquitination (Wei et al., 2024). Similar to acetylation, SUMOylation can compete with ubiquitination for the same lysine residues to regulate protein stability (Wei et al., 2024).

DROSHA has been reported to be SUMOylated at K388 and K409 in a HeLa cell high-throughput dataset (Hendriks et al., 2014); however, the function of the SUMOylations remains unknown. DGCR8, on the other hand, has been reported to be subjected to SUMOylation at K259, K426, and K707, with K259 and K707 being the primary sites of SUMO attachment (Li et al., 2023; Zhu et al., 2016; 2015). SUMO1-conjugation at DGCR8 K259 prevents DGCR8 from nuclear export and confers tumor suppression function (Zhu et al., 2016). This modification is promoted by the tumor suppressor protein p14ARF that primarily functions by binding and sequestering MDM2 to prevent the downstream MDM2-mediated protein degradation (Cilluffo et al., 2020; Weber et al., 1999). Conversely, SUMO1 modification at DGCR8 K707 promotes tumorigenesis and metastasis (Zhu et al., 2015). This SUMOylation is promoted by the ERK-mediated DGCR8 phosphorylation, antagonizing DGCR8 ubiquitination and thus enhancing its protein stability (Zhu et al., 2015). It also reinforces the affinity of DGCR8 for its pri-miRNA substrates without affecting downstream processing (Zhu et al., 2015). DGCR8 can also be modified by SUMO2 at all three SUMO-susceptible lysine residues, with the SUMOylation mediated by the deubiquitinase USP36 (Li et al., 2023). USP36-mediated SUMOylation promotes DGCR8’s affinity towards its pri-miRNA substrates, as well as promotes cell proliferation without altering its expression and its interaction with DROSHA (Li et al., 2023).

## 4 Conclusions and perspectives

### 4.1 Summary of key insights

MiRNAs are important regulators of gene expression, facilitating different cellular processes through mediating mRNA degradation or translational repression. Consistent with their importance, their synthesis is tightly regulated at multiple levels, including transcription, splicing, and RNA modifications (Kim et al., 2025). Beyond the transcriptional regulation of the miRNA genes themselves, the miRNA machinery and their associated proteins are also strictly regulated to fine-tune the miRNA production, with PTMs playing an essential role in the modulation.

In this review, we have summarized the reported PTMs of the Microprocessor, which modulate the Microprocessor in multiple ways, from its expression to substrate affinity, as well as its protein-protein interactions. These PTMs can profoundly influence the canonical miRNA biogenesis and other non-canonical Microprocessor activities, including mRNA cleavage, mRNA alternative splicing, and DNA repair. Disruption of DROSHA or DGCR8 expression impairs miRNA production and contributes to developmental defects, immune disorders, and neurological diseases, while upregulation of DROSHA and DGCR8 can enhance proliferation, promoting tumorigenesis.

### 4.2 Current limitations and challenges

Although significant progress has been made in identifying PTMs, most reported sites have been identified primarily through high-throughput proteomic discovery mass spectrometry, with little or no functional investigation. Moreover, many studies often rely on the overexpression of tagged proteins in cell lines, and thus, identified PTMs may not necessarily be physiologically relevant. Where functions of specific PTMs have been assessed, these again have often been studied in cell lines. In the future, it will be important to address function by genetic manipulation of the endogenous proteins and to study the function of these PTMs *in vivo*, such as with mouse models.

Another challenge is that most PTMs are reversible and highly dynamic, making them difficult to be detected in real time. For instance, K48-linked ubiquitinated proteins are rapidly degraded via the proteasomal pathway and thus are hard to be captured and have the corresponding modified sites identified. Moreover, the SUMO1 DGCR8 SUMOylation examples indicate that the same type of modification can exert counteracting effects. Whereas the phosphorylation of DROSHA S300 participates in different phosphorylation patterns to control opposing translocations, highlighting the site-specific and collaborative nature of PTM regulation. Furthermore, PTMs sometimes function sequentially or even antagonistically. For example, DGCR8 phosphorylation facilitates SUMOylation; acetylation and ubiquitination compete on the same lysine residues in DROSHA. These complications make functional characterization technically challenging with current PTM-mimetic and -deficient mutant approaches, which can barely resolve the complicated crosstalk between PTMs.

### 4.3 Future directions

Addressing these challenges will require more sophisticated approaches and efforts to identify Microprocessor PTMs, elucidate their functions, and dissect the complexitiy of crosstalk between specific PTMs. Targeting PTMs on the Microprocessor or the upstream enzymes may provide strategies to restore the abnormal expression level and activity of the Microprocessor in diseases and potentially alleviate disease progression. Together, these efforts will deepen our understanding of RNA biology and offer novel therapeutic opportunities for disease diagnosis and intervention.
